# Identification of Metabolite Biomarkers Associated With Dietary Patterns in Individuals With Mild Cognitive Impairment and Dementia From Yucatan

**DOI:** 10.1002/fsn3.70859

**Published:** 2025-08-28

**Authors:** Angel Gabriel Garrido‐Dzib, Berenice Palacios‐González, Erandi Bravo‐Armenta, Azalia Avila‐Nava, Roberto Lugo, Ana Ligia Gutiérrez‐Solis

**Affiliations:** ^1^ Hospital Regional de Alta Especialidad de la Península de Yucatán, Servicios de Salud del Instituto Mexicano del Seguro Social para el Bienestar (IMSS‐BIENESTAR) Mérida Yucatán México; ^2^ Facultad de Medicina Universidad Autónoma de Yucatán Mérida Yucatán México; ^3^ Laboratorio de Envejecimiento Saludable del Instituto Nacional de Medicina Genómica (INMEGEN) Centro de Investigación Sobre el Envejecimiento Ciudad de México México; ^4^ Dirección de Investigación Instituto Nacional de Medicina Genómica (INMEGEN) Ciudad de México México

**Keywords:** dementia, dietary pattern, metabolomics, Mexico, mild cognitive impairment, older adults

## Abstract

Many countries have experienced a dietary transition marked by an increased consumption of ultra‐processed foods, which is associated with poorer cognitive function. Our research group has previously identified the dietary patterns of patients with MCI and dementia, showing a significant nutritional deficiency, as the intake of vegetables, fruits, and proteins was inadequate. The identification of early biomarkers, such as metabolites associated with dietary patterns, in individuals with MCI and dementia in Mexico has not yet been explored. These metabolites could provide insights into the nutritional response or changes in individuals with these conditions and may serve as targets for early intervention. To address this gap, the aim of this study was to investigate the metabolite profiles of older adults, comparing those with neurocognitive impairments (cases) to those without (controls), based on their dietary patterns. A cross‐sectional study was carried out among 39 patients as controls and 34 individuals as cases (MCI and dementia). Concentrations of serum acylcarnitines, free carnitine, and amino acids were measured using the approach of targeted metabolomics by electrospray tandem mass spectrometry and evaluated through partial least‐squares discriminant analysis (PLS‐DA). Six metabolites “Ornithine, C18OH, Alanine, C8, C10, and C5” were identified for individuals with MCI and dementia. Levels of ornithine were correlated to the consumption of pastries and cookies (*r*_s_ = 0.33, *p* = 0.028). Three metabolites “valine, tyrosine, and methionine” were identified in the control group. Higher levels of methionine were positively correlated with vegetables intake (*r*_s_ = 0.298, *p* = 0.003). We found that certain metabolites associated with dietary patterns of MCI and dementia subjects from Yucatan, Mexico may influence cognitive status.

## Graphical abstract

Certain metabolites associated with dietary patterns of MCI and dementia subjects may influence cognitive status. Levels of ornithine were correlated to the consumption of pastries and cookies in MCI and dementia subjects. Higher levels of methionine were positively correlated with vegetables intake in older adults.

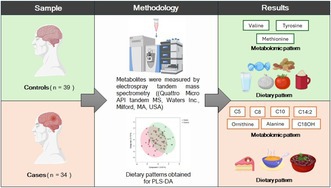

**Abbreviations**
AD: Alzheimer's disease
BCAA: branched‐chain amino acid
C12: dodecanoylcarnitine
C12:1: dodecenoylacylcarnitine
C14:2: tetradecadienoylcarnitine
C18OH: 3‐hydroxystearoylcarnitine
C4: butylcarnitine
C5: isovalerylcarnitine
C6: hexanoylcarnitine
DASH: dietary approaches to stop hypertension
GC–MS: gas chromatography coupled to mass spectrometry
MCI: mild cognitive impairment
MIND: Mediterranean‐DASH Diet Intervention for Neurodegenerative Delay
MNA‐SF: mini nutritional assessment‐short form
PLS‐DA: partial least‐squares discriminant analysis
SFFQ: semi‐quantitative food frequency questionnaire
T2D: type 2 diabetes
VD: vascular dementia
VIP: variable importance in the projection

## 1 Introduction

The global aging population is on the rise, and in Mexico, approximately 16 million residents are 60 and older (*Instituto Nacional de Estadística y Geografía (INEGI). Estadísticas a Propósito del Día Internacional de las Personas de Edad Datos Nacionales* 2022). Notably, mild cognitive impairment (MCI) and dementia are among the most prevalent health problems linked to aging (“2021 Alzheimer's disease facts and figures” 2021; Albert et al. 2011). Both conditions are characterized by cognitive decline, behavioral changes, and impaired reasoning, among other symptoms (Petersen et al. 2014, 2018). However, it's crucial to recognize that dementia symptoms are severe enough to disrupt daily life significantly, whereas individuals with MCI can maintain their independence despite some cognitive challenges (Knopman and Petersen 2014).

Dementia is a leading cause of disability among older adults, posing a significant burden not only on healthcare systems at a macro level but also on families who serve as primary caregivers at a micro level (Gutiérrez‐Robledo and Arrieta‐Cruz 2015). Alzheimer's disease (AD) is the most commonly diagnosed type of dementia, accounting for 70% of cases (“Estimation of the global prevalence of dementia in 2019 and forecasted prevalence in 2050: an analysis for the Global Burden of Disease Study 2019” Nichols et al. 2022). In recent decades, Mexico has seen one of the highest prevalences of type 2 diabetes (T2D), hypertension, and obesity—conditions that are strongly associated with both AD and vascular dementia (VD) (“Instituto Nacional de Salud Pública, Instituto Nacional de Estadística y Geografía. Base de datos y cuestionario para ENSANUT 2018” 2019).

Scientific evidence indicates that adhering to a healthy lifestyle, which includes healthful dietary habits, regular physical activity, and avoiding alcohol and tobacco, is associated with better cognitive function and brain health (Dominguez et al. 2021; Qi et al. 2023). Additionally, making healthier choices can help regulate glucose and lipid levels, as well as blood pressure.

Many countries have experienced a dietary transition marked by an increased consumption of ultra‐processed foods, which is associated with poorer cognitive function (Marrón‐Ponce et al. 2019). It is well established that individuals who follow dietary patterns such as the Mediterranean diet, the Dietary Approaches to Stop Hypertension (DASH), or the Mediterranean‐DASH Diet Intervention for Neurodegenerative Delay (MIND) diets have a lower risk of developing dementia (van den Brink et al. 2019). Our research group has previously identified the dietary patterns of patients with MCI and dementia in Yucatán, Mexico. These patterns showed significant nutritional deficiencies, as the intake of vegetables, fruits, and proteins was inadequate. In contrast, older adults without cognitive decline had more varied diets that included daily servings of fruits and vegetables (Garrido‐Dzib et al. 2024).

Currently, there is no cure for or treatment to reverse dementia. Patients with MCI and dementia have lower survival rates and higher mortality compared to individuals without cognitive impairment. Additionally, poor nutritional status significantly contributes to increased mortality (Ono et al. 2023). Therefore, a nutrimetabolomics approach can be used to identify metabolites that may serve as potential dietary indicators or biomarkers, reflecting not only nutritional status but also the patient's clinical condition.

There exists a knowledge gap regarding the metabolic pathways involved in patients with MCI and dementia. The identification of early biomarkers, such as metabolites associated with dietary patterns, in individuals with MCI and dementia in Mexico has not yet been explored. These metabolites could provide insights into the nutritional response or changes in individuals with these conditions and may serve as targets for early intervention.

To address this gap, the aim of this study was to investigate the metabolite profiles of older adults, comparing those with neurocognitive impairments (cases) to those without (controls), based on their dietary patterns.

## 2 Methods

**FIGURE 1:**
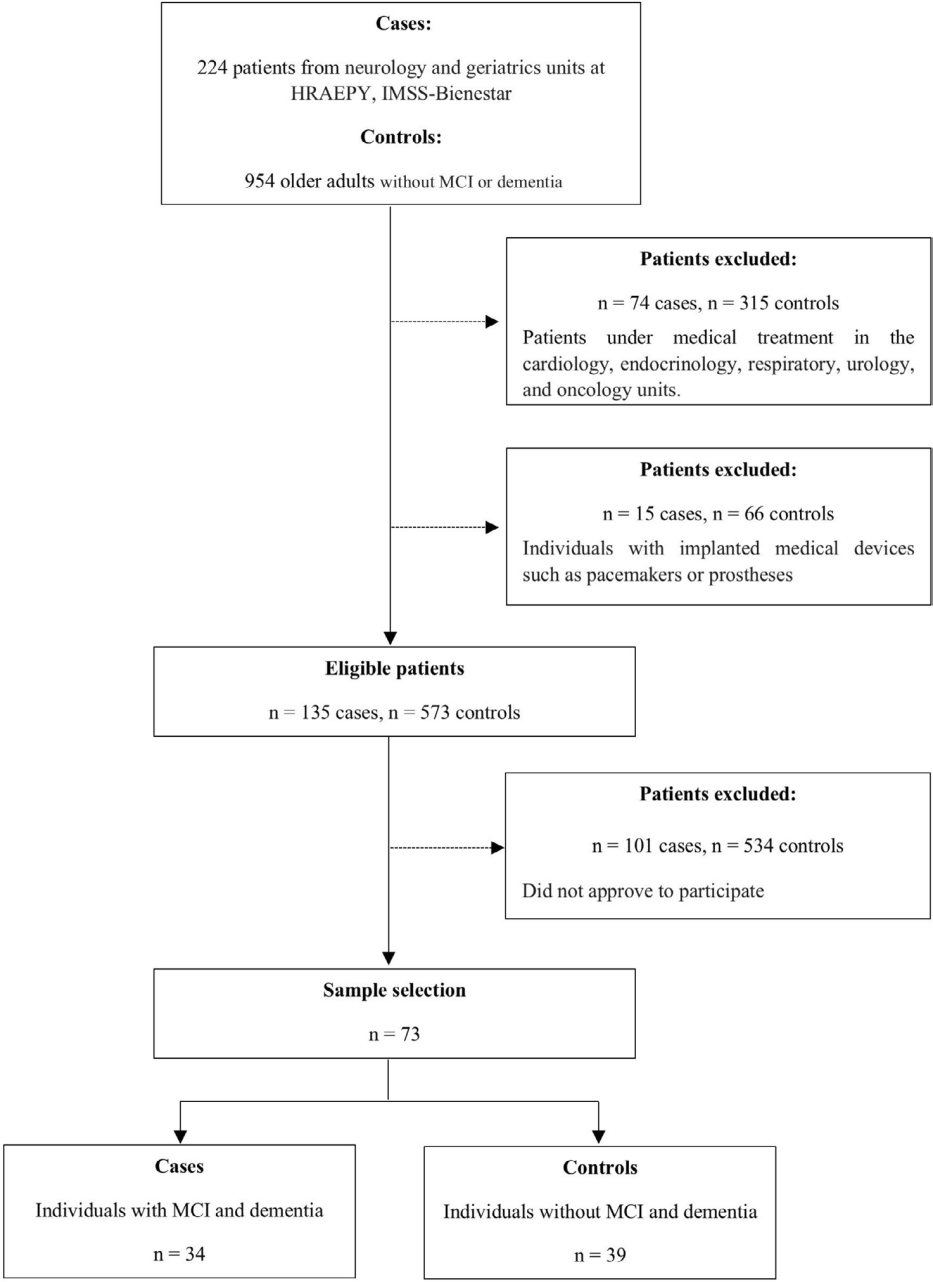
Participant flowchart of the study.

This cross‐sectional study was conducted at the Regional High Specialty Hospital of the Yucatán Peninsula, IMSS‐BIENESTAR, in Mérida, Yucatán. A total of 73 participants were included (39 controls and 34 cases). Details of the sampling procedure have been previously described and published elsewhere (Garrido‐Dzib et al. 2024). In Figure 1, the participant flowchart is detailed. The exclusion criteria encompassed participants undergoing medical treatment for various conditions in the cardiology, endocrinology, and oncology departments, as well as individuals with any type of prosthesis (medical, dental, etc.). However, participants with metabolic conditions such as T2D, hypertension and lipid alterations that were under medical and pharmacotherapy treatment were included. The study was approved by the Ethics Committee of the Regional High Specialty Hospital of the Yucatán Peninsula (no. CONBIOETICA‐31‐CEI‐002‐20170731) in association with a research project (ID Code 2021‐012), in compliance with the Declaration of Helsinki guidelines for medical research involving human subjects (“World Medical Association Declaration of Helsinki: ethical principles for medical research involving human subjects” 2013). All participants provided written informed consent.

### 2.1 Clinical Characteristics

A pre‐validated questionnaire was used to collect a brief clinical history of the participants, such as age (mean ± standard deviation), sex (women), education levels (grouped: 0–6, 7–9, and ≥ 10 years of education duration), and physical activity (categorized: ≥ 150 and < 150 min per week). The nutritional status of participants was determined using the Mini Nutritional Assessment‐Short form (MNA‐SF) and classified into malnourished‐at risk of malnutrition (0–11 points) and normal nutritional status (12–14 points).

### 2.2 Metabolomic Analysis

Concentrations of serum acylcarnitine's, free carnitine, and amino acids were measured using the approach of targeted metabolomics by electrospray tandem mass spectrometry (Quattro Micro API tandem MS, Waters Inc., Milford, MA, USA). Metabolite levels in serum were analyzed using the commercial kit (NeoBase Nonderivatized MS/MS Kit, Perkin Elmer, Waltham, MA, USA). In brief, 20 μL of serum samples were dropped onto filter paper cards (Whatman 903, Schleicher and Schüell, Dassel, Germany) and dried for 4 h at room temperature in a sterile environment. The resulting spot was precisely cut off in 2 mm circles and placed into a 96‐well plate, and then 190 μL of extraction solution containing a mixture of 22 stable isotope‐labeled internal standards was added. The plate was sealed, incubated under stirring (30°C at 650 × *g* for 30 min), and placed in a Waters autosampler. An HPLC pump (Waters 2795) was employed for the delivery of solvent, supplying a 0.1 mL/min stream of a mixture of acetonitrile: water (80:20 v:v%). Ten microliters of each sample were directly administered into the flow at 4‐min intervals. A blank sample containing extraction solution and internal standards was included in each plate in triplicate as a reference. A MicromassQuattro instrument (Waters Inc., Milford, MA, USA) coupled to an ESI source in positive mode was employed. For desolvation and nebulization, nitrogen gas was utilized, while argon was employed as the collision gas (De Jesús et al. 2010; Palacios‐González et al. 2021).

### 2.3 Dietary Patterns

Details of the dietary assessment have been previously described and published elsewhere (Garrido‐Dzib et al. 2024). Briefly, a Mexican‐validated semi‐quantitative food frequency questionnaire (SFFQ) was used to collect data on daily diet over the previous 7 days. Nineteen food groups were identified and analyzed. The food groups that showed discrimination between groups and were classified into the dietary patterns of MCI and dementia individuals were “pastries and cookies,” “soups,” and “legumes.” The dietary pattern of older adults without cognitive impairment was characterized by the following food groups: “nuts and seeds,” “candies,” “vegetables,” “coffee and tea,” and “water” with an accuracy of 0.69, R2: 0.354, Q2: 0.108, and permutation *p* value < 0.005.

### 2.4 Statistics

Descriptive statistics were calculated for the control and cases (MCI and dementia) groups. The Shapiro–Wilk test was used to check the normality. Baseline characteristics such as education, nutritional status, and physical activity were presented as proportions and corresponding percentages (%) and tested by Pearson's chi‐squared test. Age was presented as means ± standard deviation (SD) and compared using the independent *t*‐test. A Spearman correlation (*r*_s_) analysis assessed the relationship between group foods and serum metabolites. For all analyses, statistical significance was set at *p* < 0.05. The statistical packages Jamovi (version 2.25, Sydney, Australia) and SPSS version 15.00 (IBM Corp, Armonk, New York, United States) were used to analyze the data.

Partial least‐squares discriminant analysis (PLS‐DA) was employed to detect metabolite discrimination using the web‐based tool Metaboanalyst 5.0 (24). Row‐wise, normalization (normalization to constant sum), data transformation (cubic root transformation), and data scaling (Pareto scaling) were the approaches chosen for metabolite patterns. Permutation testing was conducted to minimize the possibility that the observed separation on PLS‐DA was by chance. Additionally, for cross‐validation (R2, Q2, and accuracy), model validation was performed using a 2000 times permutation test. To identify the metabolites that differentiated the groups, a loading scatter plot was created. The variable importance in the projection (VIP) plot was performed based on their significance in discerning studies from both groups. VIP cutoff > 1.0 was designated since the number of variables in this study was less than 100.

## 3 Results

The clinical characteristics of the study population have been described elsewhere. The control group had a higher level of education, with 54% having completed 10 or more years of schooling, compared to only 26.5% of the cases. Regarding nutritional status, 60% of cases reported being malnourished or at risk of malnutrition, and 69% of the participants in the control group showed themselves to have a normal nutritional status. There were no differences in the frequency of physical activity among groups in Table 1.

**TABLE 1:** Baseline characteristics among controls (*n* = 39) and cases (*n* = 34).

Characteristics	Controls (*n* = 39)	Cases (*n* = 34)	*p*
Age (years)	67.2 ± 6.52	73.4 ± 10.1	**0.002**
Women, (%)	30 (76.9)	20 (58.8)	0.097
**Education (%)**			
0–6 years	9 (23.1)	19 (55.9)	
7–9 yeas	9 (23.1)	6 (17.6)	**0.013**
≥ 10 years	21 (53.8)	9 (26.5)	
**Nutritional status (%)**			
Malnourished – at risk of malnutrition	12 (30.8)	18 (60.0)	**0.015**
Normal nutritional status	27 (69.2)	12 (40.0)	
**Physical activity (%)**			
≥ 150 min per week	17 (43.6)	11 (32.4)	0.325
< 150 min per week	22 (56.4)	23 (67.6)	

*Note:* Data are presented using proportions and percentages (%). Differences between groups were evaluated by Pearson's chi‐squared test. Statistically significant *p*‐values (< 0.05) are highlighted in bold.

Among the 40 analyzed concentrations of metabolites, it was found that the vast majority of the acylcarnitine species presented higher concentrations in individuals with MCI and dementia; however, only butylcarnitine (C4) (0.08 μmol/L; *p* = 0.031), isovalerylcarnitine (C5) (0.06 μmol/L; *p* = 0.003), hexanoylcarnitine (C6) (0.10 μmol/L; *p* = 0.001), dodecanoylcarnitine (C12) (0.30 μmol/L; *p* = 0.007), dodecenoylacylcarnitine (C12:1) (0.09 μmol/L; *p* = 0.002), tetradecadienoylcarnitine (C14:2) (0.04 μmol/L; *p* = 0.004), and 3‐hydroxystearoylcarnitine (C18OH) (0.009 μmol/L; *p* = 0.014) were statistically significant. For controls, no metabolites were statically elevated in concentrations in Table 2.

**TABLE 2:** Baseline characteristics among controls (*n* = 39) and cases (*n* = 34).

Metabolite	Controls (*n* = 39)	95% CI	Cases (*n* = 34)	95% CI	*p*
ARG	14.23 ± 3.58	13.1–15.4	15.22 ± 4.10	13.9–16.6	0.270
CIT	11.5 ± 2.33	10.8–12.3	12.8 ± 6.69	10.6–15.1	0.486*
GLY	97.3 ± 28.68	88.3–106	100.6 ± 24	92.6–109	0.364*
ALA	141.7 ± 29.53	132–151	153 ± 44.73	138–168	0.293*
LEU	48.50 ± 11.91	44.8–52.2	50.03 ± 12.30	45.9–54.2	0.592
MET	2.97 ± 0.54	2.80–3.14	2.74 ± 0.54	2.56–2.93	0.085
PHE	17.5 ± 3.93	16.3–18.8	19 ± 5.49	17.2–20.9	0.248*
TYR	21.51 ± 5.20	19.9–23.2	19.80 ± 4.88	18.2–21.5	0.154
VAL	52.61 ± 12.70	48.6–56.6	49.60 ± 12.22	45.5–53.7	0.308
ORN	7.00 ± 1.70	6.47–7.54	7.86 ± 2.60	6.99–8.74	0.098
PRO	70.3 ± 24.5	62.7–78.1	74.4 ± 25.5	65.9–83.0	0.406*
SA	1.92 ± 0.11	1.89–1.96	1.95 ± 0.10	1.92–1.99	0.246
C0	18.43 ± 3.96	17.2–19.7	18.90 ± 4.62	17.3–20.5	0.645
C2	0.03 ± 0.012	0.02–0.04	0.03 ± 0.018	0.03–0.03	0.410*
C3	0.01 ± 0.004	0.011–0.014	0.01 ± 0.005	0.012–0.016	0.461*
C6DC	1.48 ± 0.07	1.46–1.51	1.50 ± 0.06	1.48–1.53	0.278
C4	0.07 ± 0.01	0.070–0.078	0.08 ± 0.02	0.07–0.09	0.031*
C5	0.05 ± 0.015	0.05–0.06	0.06 ± 0.12	0.05–0.06	0.003*
C5:1	0.04 ± 0.008	0.035–0.041	0.04 ± 0.009	0.036–0.042	0.676*
C6	0.09 ± 0.01	0.08–0.09	0.10 ± 0.01	0.09–0.10	**0.001**
C8	0.03 ± 0.008	0.031–0.036	0.04 ± 0.02	0.035–0.052	0.034*
C8:1	0.05 ± 0.01	0.043–0.058	0.05 ± 0.01	0.0496–0.060	0.995*
C16	0.04 ± 0.01	0.038–0.042	0.04 ± 0.01	0.035–0.044	0.510*
C16:1	0.01 ± 0.005	0.012–0.015	0.01 ± 0.006	0.013–0.017	0.455*
C16:1OH	0.01 ± 0.03	0.009–0.010	0.01 ± 0.001	0.009–0.010	0.552*
C16OH	0.04 ± 0.005	0.042–0.045	0.05 ± 0.006	0.044–0.048	0.128*
C10	0.06 ± 0.01	0.059–0.071	0.08 ± 0.04	0.066–0.099	0.095*
C10:1	0.39 ± 0.03	0.388–0.410	0.40 ± 0.04	0.390–0.419	0.531
C10:2	0.03 ± 0.008	0.030–0.035	0.03 ± 0.005	0.033–0.037	0.265*
C12	0.28 ± 0.02	0.277–0.293	0.30 ± 0.31	0.293–0.313	**0.007**
C12:1	0.09 ± 0.01	0.086–0.094	0.09 ± 0.01	0.095–0.184	**0.002**
C14	0.02 ± 0.004	0.016–0.020	0.02 ± 0.005	0.016–0.020	1.00*
C14:1	0.04 ± 0.00	0.045–0.051	0.05 ± 0.00	0.047–0.054	0.215
C14:2	0.03 ± 0.007	0.032–0.037	0.04 ± 0.007	0.038–0.043	0.004*
C14OH	0.01 ± 0.00	0.010–0.010	0.009 ± 0.001	0.009–0.010	0.297*
C18	0.02 ± 0.004	0.019–0.021	0.02 ± 0.008	0.018–0.024	0.676*
C18:1	0.04 ± 0.009	0.039–0.045	0.04 ± 0.017	0.036–0.045	0.327*
C18:1OH	0.005 ± 0.005	0.0038–0.006	0.005 ± 0.005	0.004–0.007	0.676*
C18:2	0.03 ± 0.007	0.028–0.033	0.02 ± 0.009	0.026–0.032	0.366*
C18OH	0.007 ± 0.004	0.005–0.008	0.009 ± 0.002	0.008–0.010	0.014*

*Note:* Data are presented using mean ± standard deviation. Differences between groups were evaluated with independent t‐test or Mann–Whitney test marked with *. Statistically significant *p*‐values (< 0.05) are highlighted in bold.

Abbreviations: 95% CI, 95% confidence intervals; C0, free carnitine; C10, decanoylcarnitine; C10:1, decenoylcarnitine; C10:2, decadienoilcarnitine; C12, dodecanoylcarnitine; C12:1, dodecenoylcarnitine; C14, myristoylcarnitine; C14:1, tetradecenoylcarnitine; C14:2, tetradecadienoylcarnitine; C14OH, 3‐Hydroxytetradecanoylcarnitine; C16, palmitoylcarnitine; C16:1, hexadecenoylcarnitine; C16:1OH, 3‐Hydroxypalmitoleylcarnitine; C16OH, 3‐hidroxi‐palmitoilcarnitine; C18, acylcarnitine; C18:1, oleic acid; C18:1OH, 11‐hydroxy oleic acid; C18:2, linoleylcarnitine; C18OH, 3‐hydroxystearoylcarnitine, 18‐hydroxyoleic acid; C2, acetylcarnitine; C3, propionylcarnitine; C4, butylcarnitine; C5, isovalerylcarnitine; C5:1, tiglylcarnitine; C6, hexanoylcarnitine; C6DC, adipoylcarnitine; C8, octanoylcarnitine; C8:1, octenoylcarnitine.

**FIGURE 2:**
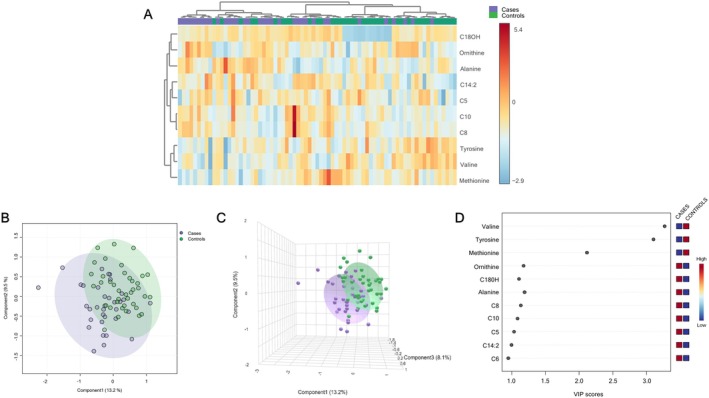
Metabolite patterns are profiled according to the group of cases (MCI and dementia) and control. (A) Hierarchical heatmap for dietary patterns: control (green) and cases (purple); red and blue show increasing and decreasing food group consumption, respectively. (B) The 2D score plot from the PLS‐DA shows separation between cases (purple) and controls (green). The explained variances are shown in brackets (accuracy: 0.634; R2: 0.367; Q2: 0.051; permutation *p* value = 0.025). (C) The 3D score plot from the PLS‐DA shows separation between cases (purple) and controls (green). (D) The VIP analysis represents the relative contribution of each metabolite to the variance among groups. A high VIP score indicates a greater contribution of the metabolite to the pattern profile. Red and blue boxes on the right indicate whether metabolite concentration is increased (red) or decreased (blue).

**FIGURE 3:**
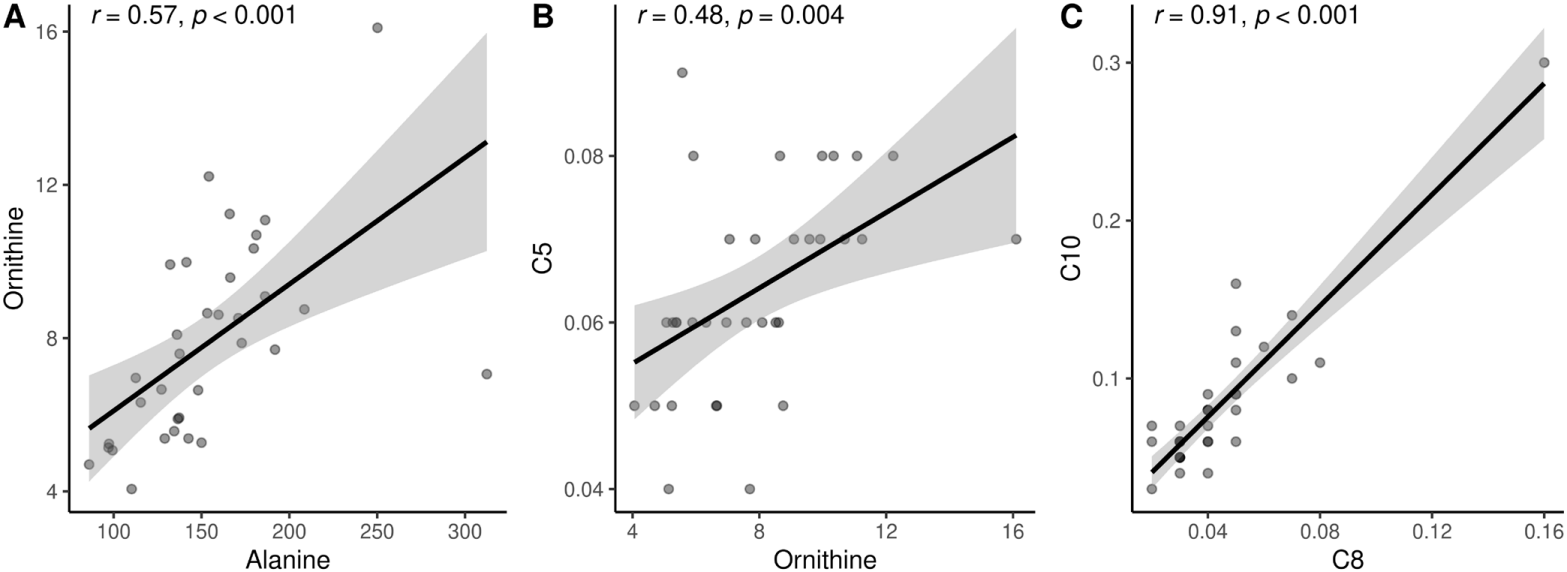
Statistical correlations among metabolites in the group of cases. (A) Alanine and ornithine. (B) Ornithine and C5. (C) C8 and C10.

**FIGURE 4:**
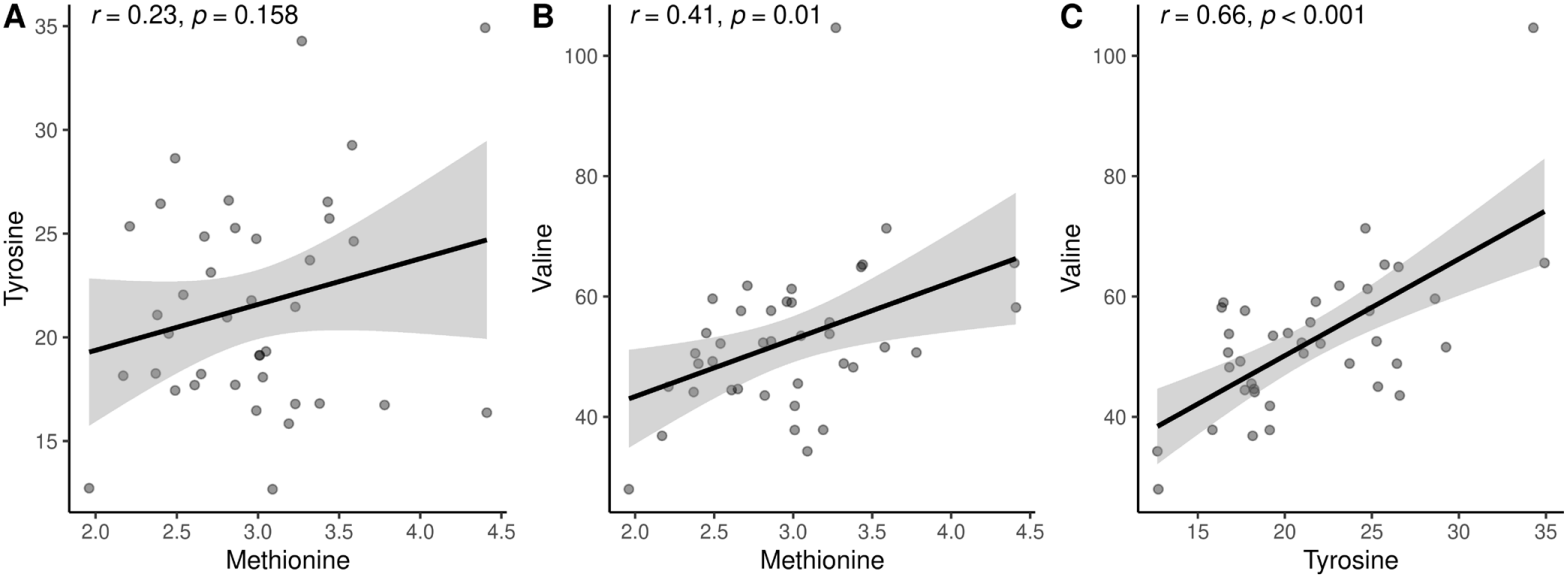
Statistical correlations among metabolites in the group of controls. (A) Methionine and tyrosine. (B) Methionine and valine. (C) Tyrosine and valine.

Serum metabolite profile was performed to visualize difference in the patterns among groups using the PLS‐DA score plots; it was found a slight evidence of separation among population with MCI and dementia and older adults in Figure 2B, an accuracy of 0.634, R2: 0.367, Q2: 0.051, and permutation *p* = 0.025. A pattern depending on having a neurocognitive condition was observed and characterized by the following metabolites “Ornithine,” “C18OH,” “Alanine,” “C8,” “C10,” and “C5.” The metabolites that were found to be in charge of differentiation in the control group included “Valine,” “Tyrosine,” and “Methionine” in Figure 2C. In Figure 3, group‐specific correlation analyses were performed for the top discriminatory metabolites. In the MCI and dementia group, positive and significant correlations were observed between Alanine and Ornithine (*r*_s_ = 0.508, *p* = 0.005), Ornithine and C5 (*r*_s_ = 0.479, *p* = 0.004), and C8 and C10 (*r*_s_ = 0.90, *p* < 0.001) among MCI and dementia. In the control group in Figure 4, significant positive correlations were found between Methionine and Valine (*r*_s_ = 0.41, *p* = 0.010) and Tyrosine and Valine (*r*_s_ = 0.66, < 0.001). No other significant correlations were observed among the remaining metabolites (Tables S1 and S2).

**FIGURE 5:**
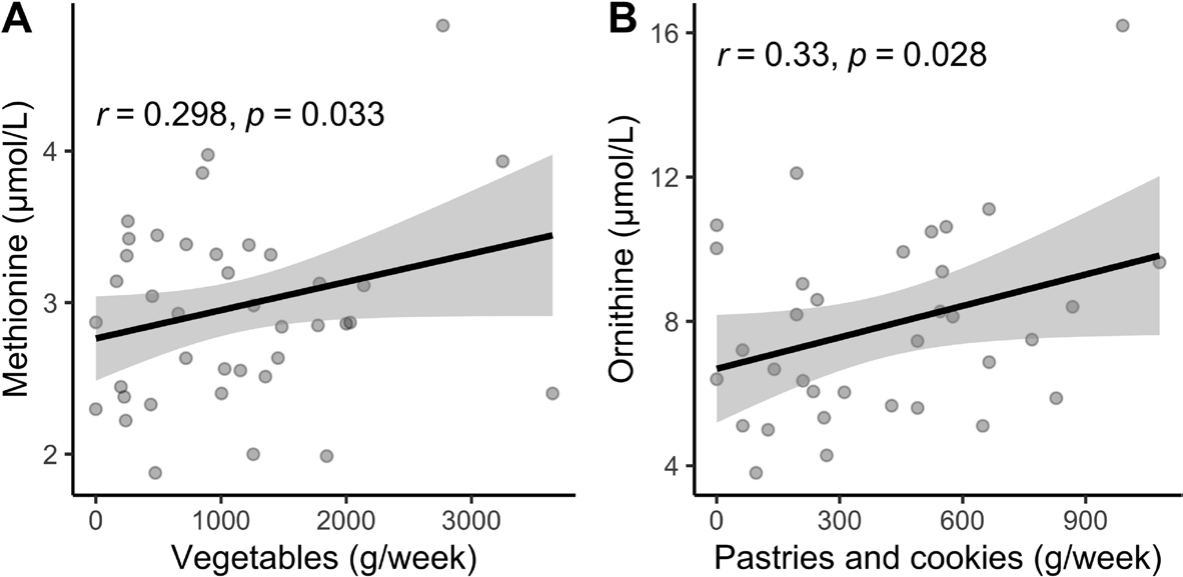
(A) Statistical correlations of vegetable consumption with methionine levels in controls; (B) Statistical correlations of pastries and cookies consumption with ornithine levels in cases.

Previous published results from our group showed that the dietary patterns of individuals with MCI and dementia were characterized by “pastries and cookies,” “soups,” and “legumes.” In contrast, the dietary pattern of older adults without cognitive impairment was characterized by “nuts and seeds,” “candies,” “vegetables,” “coffee and tea,” and “water.” Therefore, additional correlations between the metabolite profiles and dietary patterns were performed in Figure 5 and Tables 3 and 4. The results showed that the food group “vegetables,” which was part of the dietary pattern of the control group, was significantly correlated with higher levels of methionine (*r*_s_ = 0.298, *p* = 0.003), indicating that older adults who consumed vegetables had higher levels of methionine. Moreover, the dietary pattern of individuals with MCI and dementia, which was characterized by the food group “pastries and cookies,” was significantly correlated with higher levels of ornithine (*r*_s_ = 0.33, *p* = 0.028), suggesting that individuals with cognitive impairment that consumed pastries and cookies had higher serum concentrations of ornithine.

**TABLE 3:** Spearman correlation coefficient for the relationship among metabolites and dietary pattern in controls (*n* = 39).

Correlation	Methionine	Tyrosine	Valine
Vegetables	**0.29**	0.13	0.11
Candies	0.12	−0.00	−0.01
Nuts and seeds	0.19	0.08	0.13
Water	−0.09	**0.29**	0.19
Coffee and tea	−0.02	−0.09	**0.03**

*Note:* Statistically significant *p*‐values (< 0.05) are highlighted in bold.

**TABLE 4:** Spearman correlation coefficient for the relationship metabolites and dietary pattern in cases (*n* = 34).

Correlation	Alanine	Ornithine	C5	C8	C10
Legumes	−0.11	0.13	0.09	−0.21	−0.23
Pastries and cookies	0.16	**0.33**	0.08	−0.22	−0.29
Soups	0.04	−0.09	−0.07	−0.26	−0.23

*Note:* Statistically significant *p*‐values (< 0.05) are highlighted in bold.

## 4 Discussion

This study included 73 individuals, comprising 34 individuals with MCI and dementia, and 39 control participants without cognitive decline. We identified six metabolites—Ornithine, C18OH, Alanine, C8, C10, and C5—corresponding to individuals with MCI and dementia. Among them, levels of ornithine were correlated to the consumption of pastries and cookies.

Furthermore, the individuals with MCI and dementia also exhibited significantly fewer years of education and were characterized by a nutritional status indicating they were malnourished or at risk of malnutrition.

Three metabolites—valine, tyrosine, and methionine—were identified in the control group. Additionally, higher levels of methionine were positively correlated with vegetable intake.

Comprehending the relationship between diet and dementia‐related metabolites provides valuable insights into the biochemical mechanisms through which nutrition influences cognitive health.

Ornithine and alanine are amino acids; however, they have different metabolic functions. Ornithine is linked to the urea cycle and plays the role of eliminating ammonia from the body. If the urea cycle has a dysfunction, this could lead to the accumulation of ammoniac and result in damage in some organs, including the brain (Sivashanmugam et al. 2017; van der Lee et al. 2018). Scientific evidence has shown that elevated concentrations of ornithine have been associated with Parkinson's disease (PD) (Akdas et al. 2024) and dementia (van der Lee et al. 2018). A systematic review that analyzed the serum of patients with AD and vascular dementia showed no significant differences in ornithine levels between controls and cases (Zinellu et al. 2023). Another study by Ozaki et al. evaluated levels of metabolites in plasma, including ornithine; a decreased level was found in AD patients compared to controls. However, the authors hypothesized that these low levels could result from an aberrant polyamine pathway or urea cycle (Ozaki et al. 2022). The blood metabolome reflects the consumption of specific foods, dietary patterns, and their interactions with endogenous metabolism, offering rich information on a wide range of biological activities. Our study found a positive correlation between serum ornithine levels and the consumption of pastries and cookies. These results contrast with those of a study conducted on Asian adults. Yong et al. (2023) reported significantly lower ornithine levels in subjects with high‐glycemic index food consumption (such as pastries and cookies) compared to those consuming low‐glycemic index foods.

During glycogenesis, the body consumes alanine to create glucose and energy. Elevated alanine levels indicate several metabolic issues, including infections, stress, chronic liver illnesses, and others (Paulusma et al. 2022). In this regard, Hernández et al. obtained similar results when they examined untargeted metabolites using post‐mortem human samples and validated them with the PLS‐DA model. The alanine, aspartate, and glutamate pathways and the arginine, phenylalanine, and tyrosine pathways were meaningful for separating AD patients and controls (Hernandez et al. 2025).

Valine, tyrosine, and methionine are essential amino acids; therefore, they must be obtained through the diet (Fu et al. 2024). Specifically, valine is a branched‐chain amino acid (BCAA), which mainly plays a role to obtain energy from muscles (Shimomura et al. 2006). Although it has been identified that valine regulates some neurotransmitters, its relationship with cognitive function is less direct than that of other amino acids such as leucine and isoleucine, but as part of the BCAAs, it contributes to protein synthesis and neurotransmitter balance (Brosnan and Brosnan 2006; Fernstrom 2005). A study in patients with AD that used gas chromatography coupled to mass spectrometry (GC–MS) observed a shift in the serum levels among 23 metabolites; it was found that there were decreased levels of urea, valine, aspartate, pyroglutamate, glutamine, phenylalanine, asparagine, ornithine, pipecolic acid, among others (Lista et al. 2023). In an experimental study, it was explored the potential of co‐assembling Aβ42 with its G37V variant (Aβ42 (G37V)), where glycine at position 37 was substituted with valine, showing that Aβ42 (G37V) was able to modulate Aβ42 aggregation and reduce neurotoxic effects (Huang et al. 2024).

Tyrosine is a precursor to several important neurotransmitters, including dopamine and norepinephrine (Jongkees et al. 2015), which play vital roles in attention, stress response, and memory (Daubner et al. 2011; Kvetnansky et al. 2009). Research has demonstrated that tyrosine can enhance cognitive performance by reversing the neurotransmitter depletion, especially while under stress or other cognitive demands (Brooks and Piccini 2006; Jongkees et al. 2015; Kvetnansky et al. 2009). Some studies suggest that tyrosine supplementation may improve the proactive response inhibition in subjects aged between 60 and 75 (Bloemendaal et al. 2018).

Methionine is an essential sulfur‐containing amino acid that plays a crucial role in protein synthesis (Parkhitko et al. 2019). In particular, dietary methionine is converted to homocysteine, and after synthesis, the final product generated is cysteine, which participates in the production of antioxidants and other nonessential amino acids (Xu et al. 2024). However, there are contradictory findings regarding the relationship between methionine levels and cognitive decline (Zhao et al. 2022). Some studies suggest that dietary restriction of methionine may correlate with improved cognitive function (Xi et al. 2023). For instance, Hooshmand et al. (2019) reported that elevated serum methionine levels could be associated with brain atrophy and an increased risk of dementia in older adults. In contrast, Corso et al. (2017) found no association between methionine levels and mild cognitive impairment (MCI) or Alzheimer's disease (AD). It is important to note that maintaining a proper methylation state relies on the availability of dietary precursors like methionine. This implies that dietary methionine intake is essential for preserving homeostasis, and that either supplementation or restriction can significantly impact health (Navik et al. 2021).

The model fit of our analysis was low, represented by explained variance (R2: 0.367) and predictive ability (Q2: 0.051), but had a good accuracy of 0.63 (More et al. 2022; Sharma and Paliwal 2015). Furthermore, the reported permutation (50/2000) was statistically significant (*p* = 0.025), confirming the validity of the PLS‐DA model at the 95% confidence level. Similar levels of power and significance were observed in our previously published work (Garrido‐Dzib et al. 2024). These results indicate that the identified metabolite patterns were relatively consistent and reliable; however, a larger sample size could potentially lead to a stronger association with greater statistical power.

The relationship between metabolite levels and food consumption is an emerging area of research. Some studies have begun to explore how dietary patterns, like Mediterranean and MIND diets, affect these relationships. Tanaka et al. (2022) identified four metabolites (TrpBetaine, CE(17:1), PCaa C40:5, and PCae C42:3) that were common to both the MIND and Mediterranean diets in older adults. Their findings suggest that adherence to healthy dietary patterns might slow the development of certain cognitive deficits by modulating various metabolic pathways represented by these metabolites. Additionally, the study showed a positive correlation between vegetable consumption and methionine levels, with onions being the most frequently consumed vegetable in this group. Onions possess numerous biological properties, including antibacterial, antimutagenic, and antioxidant properties, and they are rich in amino acids like cysteine, methionine, and lectins.

Given that metabolic‐related dementia diseases are surprisingly common in the Yucatán's population (Banik et al. 2022; Datta Banik et al. 2021), omics research becomes crucial. It can provide a comprehensive understanding of the molecular mechanisms underlying these conditions and help identify reliable metabolites that may be used to stratify the risk of dementia. Furthermore, interventions that incorporate local food with antioxidant properties could be beneficial in modulating the concentrations of relevant metabolites within the study population (Andrés et al. 2024; Guevara‐Cruz et al. 2021).

There are several limitations to this study. Using disparate techniques to assess nutrition and metabolites is a significant problem when comparing metabolomic studies of dietary patterns and metabolic conditions. Furthermore, due to the cross‐sectional methodology, the data we present are associations rather than causal links. Nevertheless, this is the first study to evaluate the metabolomics profile of older Mexican persons with dementia and MCI. Furthermore, the metabolites found in this study were connected to the dietary pattern established through a posteriori analysis. This is a more accurate representation of the actual diet of our population because a priori approaches typically involve pre‐established diets like the Mediterranean and MIND, which contain foods that are unavailable in our environments. Identifying and understanding the relationship between dietary patterns and metabolites, as well as the underlying mechanisms and pathways involved, will be made easier using long‐term studies.

## 5 Conclusion

We found that certain metabolites associated with dietary patterns of MCI and dementia subjects may influence cognitive status.

## Author Contributions

**Ana Ligia Gutiérrez‐Solis:** conceptualization (equal), data curation (equal), formal analysis (equal), investigation (equal), project administration (equal), resources (equal), supervision (equal), validation (equal), writing – original draft (equal), writing – review and editing (equal). **Angel Gabriel Garrido‐Dzib:** conceptualization (equal), data curation (equal), formal analysis (equal), investigation (equal), validation (equal), visualization (equal), writing – review and editing (equal). **Berenice Palacios‐González:** conceptualization (equal), data curation (equal), formal analysis (equal), methodology (equal), project administration (equal), resources (equal), supervision (equal), validation (equal), writing – original draft (equal), writing – review and editing (equal). **Erandi Bravo‐Armenta:** investigation (equal), methodology (equal), project administration (equal), supervision (equal), writing – review and editing (equal). **Azalia Avila‐Nava:** investigation (equal), project administration (equal), supervision (equal), validation (equal), writing – review and editing (equal). **Roberto Lugo:** investigation (equal), project administration (equal), validation (equal), writing – review and editing (equal).

## Conflicts of Interest

The authors declare no conflicts of interest.

## Supporting information


**Data S1:** Correlation analysis among metabolites in the case group (*n* = 34).

**Table S1:** Correlation analysis among metabolites in the case group (*n* = 34).
**Table S2:** Correlation analysis among metabolites in the control group (*n* = 39).

## Data Availability

The raw metabolite data are included in the Supporting Information. Further datasets generated and analyzed during the current study are available from the corresponding author upon reasonable request.
