# Supplementary material for: Identification of Metabolite Biomarkers Associated With Dietary Patterns in Individuals With Mild Cognitive Impairment and Dementia From Yucatan
Source: Food Sci Nutr. 2025 Aug 28;13(9):e70859. doi: 10.1002/fsn3.70859 (PMC12394731; doi:10.1002/fsn3.70859)
Supplement: Supplementary file 2 — Table S1: Correlation analysis among metabolites in the case group (n = 34). Table S2: Correlation analysis among metabolites in the control group (n = 39). [file FSN3-13-e70859-s001.docx]

**Supplementary Tables**

**Table S1**. Correlation analysis among metabolites in the case group (n=34).

| **Metabolites** | **r** | **p-value** |
| --- | --- | --- |
| Alanine-Ornithine | 0.508 | **0.005** |
| Alanine-C5 | 0.290 | 0.0959 |
| Alanine-C8 | 0.039 | 0.8286 |
| Alanine-C10 | -0.050 | 0.7796 |
| Ornithine-C5 | 0.479 | **0.0041** |
| Ornithine-C8 | 0.117 | 0.5082 |
| Ornithine-C10 | 0.042 | 0.8142 |
| C5-C8 | -0.013 | 0.9404 |
| C5-C10 | 0.011 | 0.9507 |
| C8-C10 | 0.905 | **< 0.001** |

**Table S2**. Correlation analysis among metabolites in the control group (n=39).

| **Metabolites** | **r** | **p-value** |
| --- | --- | --- |
| Methionine-Tyrosine | 0.230 | 0.1584 |
| Methionine-Valine | 0.407 | **0.0102** |
| Tyrosine-Valine | 0.658 | **< 0.001** |
